# Evidence for a novel function of Awd in maintenance of genomic stability

**DOI:** 10.1038/s41598-017-17217-0

**Published:** 2017-12-04

**Authors:** Patrizia Romani, Serena Duchi, Giuseppe Gargiulo, Valeria Cavaliere

**Affiliations:** 1 Dipartimento di Farmacia e Biotecnologie, Alma Mater Studiorum Università di Bologna, Bologna, 40126 Italy; 2 Laboratorio di Patologia Ortopedica e Rigenerazione Tissutale Osteoarticolare, Istituto Ortopedico Rizzoli, Bologna, 40136 Italy; 3 Present Address: Dipartimento di Medicina Molecolare, Università di Padova, Padova, 35131 Italy

## Abstract

The *abnormal wing discs* (*awd*) gene encodes the *Drosophila* homolog of *NME1/NME2* metastasis suppressor genes. Awd acts in multiple tissues where its function is critical in establishing and maintaining epithelial integrity. Here, we analysed *awd* gene function in *Drosophila* epithelial cells using transgene-mediated RNA interference and genetic mosaic analysis. We show that *awd* knockdown in larval wing disc epithelium leads to chromosomal instability (CIN) and induces apoptosis mediated by activation of c-Jun N-terminal kinase. Forced maintenance of Awd depleted cells, by expressing the cell death inhibitor p35, downregulates atypical protein kinase C and DE-Cadherin. Consistent with their loss of cell polarity and enhanced level of matrix metalloproteinase 1, cells delaminate from wing disc epithelium. Furthermore, the DNA content profile of these cells indicates that they are aneuploid. Overall, our data demonstrate a novel function for *awd* in maintenance of genomic stability. Our results are consistent with other studies reporting that NME1 down-regulation induces CIN in human cell lines and suggest that *Drosophila* model could be successfully used to study *in vivo* the impact of NME/Awd - induced genomic instability on tumour development and metastasis formation.

## Introduction

Genomic stability is critical for cell survival and development and several cellular mechanisms act to maintain genomic integrity^1^. Failure of these mechanisms underlies aging and can lead to malignancies such as cancer^2^ and age-related neurodegenerative diseases^3^. Chromosomal instability (CIN) is a form of genomic instability that often leads to aneuploidy, a deleterious condition characterised by copy number changes affecting part or whole chromosomes^4^. Several dysfunctions could lead to CIN^5^. Defective activity of the spindle assembly checkpoint (SAC), a signalling pathway that blocks anaphase onset in response to mis-attachment of chromosomes to the mitotic spindle, leads to CIN and aneuploidy^6^. Work in *Drosophila* showed that loss of function of SAC genes as well as loss of function of genes involved in spindle assembly, chromatin condensation and cytokinesis induce CIN^7^. More recent work in larval disc epithelia has shown that down-regulation of these genes causes apoptotic cell death trough activation of the c-Jun N-terminal kinase (JNK) pathway^8,9^. Interestingly, blocking CIN-induced apoptotic cell death induces tumourigenic behaviour including basement membrane degradation, cell delamination, tissue overgrowth and aneuploidy.

The *abnormal wing discs* (*awd*) gene encodes the *Drosophila* homolog of *NME1/2* metastasis suppressor genes. Awd is a well-known endocytic mediator whose function is required in multiple tissues during development^10^. Genetic studies showed that Awd endocytic function ensures appropriate internalisation of chemotactic signalling receptors such as platelet-derived growth factor/VEGF receptor (PVR)^11^ and fibroblast growth factor receptor (FGFR) and thus it regulates invasion and cellular motility^12^. Furthermore, this endocytic function regulates Notch receptor trafficking^13^ and is required for maintenance of epithelial integrity as it controls the turnover of adherens junction components in ovarian somatic follicle cells^14^. Consistent with the high degree of functional conservation between Awd and its mammalian counterparts, recent studies have shown a role for the NME1/2 proteins in vesicular transport^15^.

In the present work, we have extended our analysis of the functional conservation between Awd and NME1/2 proteins. Since loss of *NME1* gene function in human cell culture leads to polyploidy^16^, we have explored the role of Awd in maintenance of genomic stability. Our data show that knockdown of *awd* in wing disc cells leads to CIN and to the CIN-induced biological responses mediated through JNK activation. Furthermore, when combined with block of apoptosis, down-regulation of *awd* leads to cell delamination and aneuploidy. Thus, the results of our *in vivo* analysis show a novel function for *awd* in maintenance of genomic stability.

## Results

### Down-regulation of *awd* leads to genomic instability and cell death

**Figure 1:**
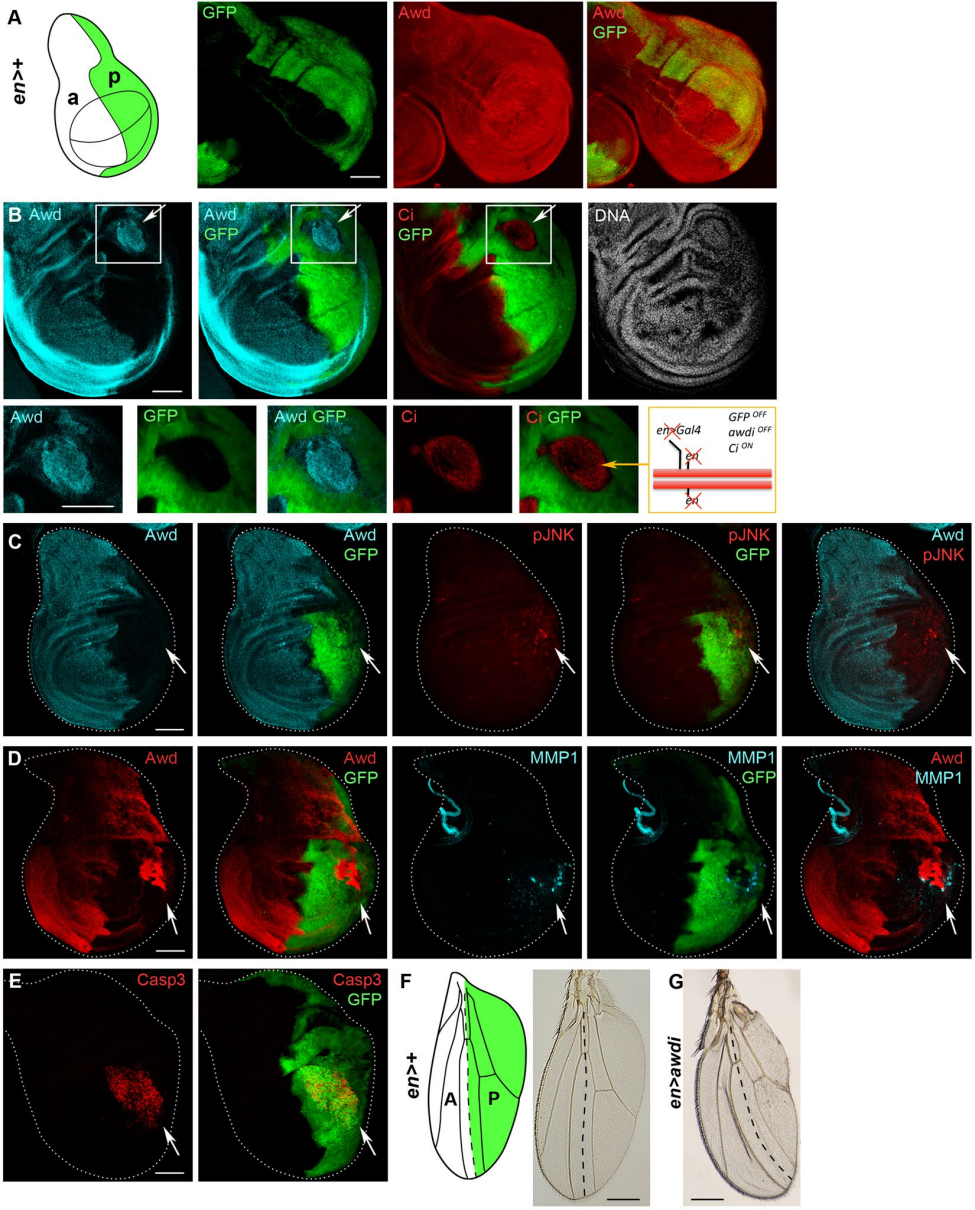
*awd* knockdown in wing discs leads to CIN. (**A**) Illustration of a third instar wing disc showing anterior (a) and posterior (p) compartments. The *en* driver posterior expression domain is coloured in green. Confocal images of wing disc expressing GFP under the control of *en* driver show that Awd protein is expressed all over the disc. (**B**–**E**) Confocal images of wing discs dissected from *en* > *awdi* larvae. (**B**) Awd and Ci double staining and DAPI staining of *en* > *awdi* wing discs. The white boxes outline the area located in the posterior region that is magnified in the bottom panels where Awd, GFP or Ci signals are shown. The scheme summarises the effect of the CIN event that occurs in the patch of cells expressing Awd and Ci, and lacking GFP expression. In the posterior region of *en* > *awdi* wing discs CIN-induced upregulation of pJNK (**C**), MMP1 (**D**) and cleaved-Caspase 3 (**E**) levels is evident. (**F**) In the illustration of the adult wing, the region that develops from the *en* posterior domain of expression in wing disc is coloured in green. In the micrographs of adult wings, dashed lines trace the boundary between anterior and posterior compartments. (**G**) In comparison with *en* > + control, wings from *en* > *awdi* adults show defects in the posterior compartment. Scale bars: 100 μm in (**A**–**E)**, 50 μm in high magnification in (**B)**, 200 μm in (**F**,**G)**.

We have analysed the effects of Awd depletion in larval wing discs since these primordia are an excellent model system to study CIN and tumourigenesis^17^. As shown in Fig. 1A, the Awd protein is expressed throughout the wing disc^13^ (n = 30). We have down-regulated *awd* expression through *UAS*/*Gal4*-driven RNA interference. The *engrailed-Gal4* (*en*) driver has been used to induce expression of the *UAS-awd-RNAi* (*awdi*) construct in the posterior compartment cells of larval wing disc (Fig. 1A). The use of the compartment specific *en-Gal4* driver allows direct comparison of wild type cells in the anterior compartment versus *awd* mutant cells in the posterior compartment within a single imaginal disc. Co-expression of the GFP marker allows easy recognition of the domain targeted for *awd* silencing in *en* > *GFP, awdi* larvae (hereafter referred as *en* > *awdi*). To mark the anterior domain, we have stained wing discs for *cubitus interruptus* (*ci*), whose expression is repressed in the posterior domain by the *en* gene product^18^. The analysis of *en* > *awdi* wing discs has shown patches of cells lacking GFP expression and expressing Awd and Ci protein despite their localisation in the posterior region of the disc where Awd is downregulated (73,3% wing discs; n = 30) (Fig. 1B). Thus, a genomic instability event has involved the second chromosome hosting the *en* driver leading to impaired *en* driver activity and loss of heterozygosity of the *UAS-GFP* and *UAS-awdi* transgenes. Furthermore, expression of the Ci anterior marker in these GFP^−^, Awd^+^ cells located in the posterior wing disc domain, demonstrates impaired *en* gene function. Thus, down-regulation of *awd* expression leads to genomic instability events that involve both copies of the second chromosome and cause loss of *en* gene function.

Further analyses of *en* > *awdi* wing discs have shown that down-regulation of *awd* in posterior domain activates JNK as indicated by the presence of the phosphorylated form of JNK (pJNK) (Fig. 1C, n = 35) and by the elevated level of matrix metalloproteinase 1 (MMP1) (Fig. 1D, n = 39), a downstream effector of JNK pathway^19^. Furthermore, these posterior cells exhibit an increased level of apoptotic death as shown by cleaved-Caspase 3 (Casp3) staining (Fig. 1E, n = 30). Genomic instability often leads to CIN and aneuploidy^4,5^. Interestingly, CIN induced by silencing of SAC genes, or downregulation of genes involved in spindle assembly and cytokinesis triggers JNK pathway activation, upregulation of MMP1 and apoptotic cell death^8,9^. Thus, our data suggest that Awd depletion leads to CIN.

Our previous studies on *awd* function already showed that this gene is required during development for normal wing development since adult flies, mosaic for *awd* ^*J2A4*^ loss of function allele, show altered wing morphology^13^. The analysis of *en* > *awdi* adults has shown that, in comparison with wild type (Fig. 1F), *en* > *awdi* wing discs develop into adult wings with a reduced posterior domain and bent toward the posterior (Fig. 1G, n = 20). This phenotype is consistent with the occurrence of apoptotic cell death induced by Awd depletion in larval wing disc.

### *awd* knockdown leads to abnormal eye differentiation

**Figure 2:**
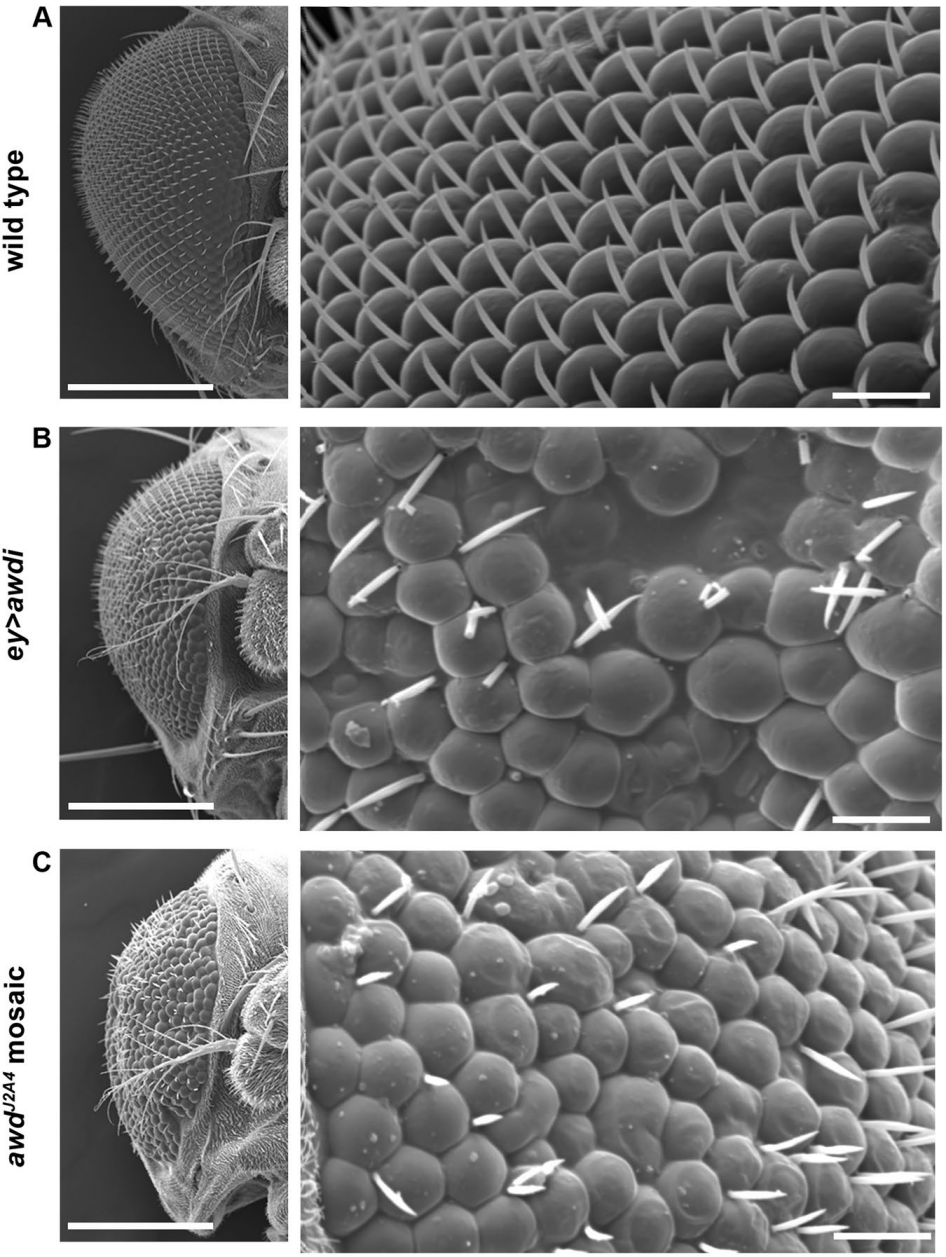
Awd depletion during development alters differentiation of adult eyes. Scanning electron microscope images of eyes (frontal view) from adult flies of the indicated genotype show severely affected morphology of *ey* > *awdi* (**B**) and *ey-flp* induced *awd* ^*J2A4*^ mosaic eyes (**C**). In comparison with wild type eye (**A**), the *awd* mutant retinae show a rough phenotype with clear defects in differentiation and growth. Scale bars: 200 μm in left panels and 20 μm in the magnifications.

We extended our analysis of *awd* gene function to the development of adult eye. The detailed analysis of the eye of adult flies expressing *awdi* in the larval eye disc under the control of the *eyeless-Gal4* driver^20^ (*ey* > *awdi*) has showed aberrant morphological phenotype. In wild type adult eye about 700 ommatidia, each with an inter-ommatidial bristle, are organised in a highly regular array (Fig. 2A). On the contrary, *ey* > *awdi* adult eyes show clear defects in alignment of ommatidial facets and bristles and with some ommatidia missing (Fig. 2B). Furthermore, we have analysed the effects of *awd* ^*J2A4*^ null mutation on eye development. Since this allele is lethal in homozygous condition, we have applied the directed mosaic technique to induce *awd* ^*J2A4*^ mosaic clones in the eye disc^21^. We have used the *ey-flp* line^22^ to target FRT/Flp site specific recombination to the eye disc. Morphological analysis of *ey-flp*-induced *awd* ^*J2A4*^ mosaic adult eyes also shows that a rough eye phenotype results from loss of *awd* function (Fig. 2C). Overall our results show that knockdown as well as complete loss of *awd* function in larval wing and eye discs impairs normal development of the corresponding adult structures. Altered differentiation of adult structures arises from CIN induced aneuploidy in wing and eye discs^8^, so our results on the role of *awd* in maintenance of genomic stability suggest that Awd depletion leads to CIN.

### *awd* silencing leads to aneuploidy

**Figure 3:**
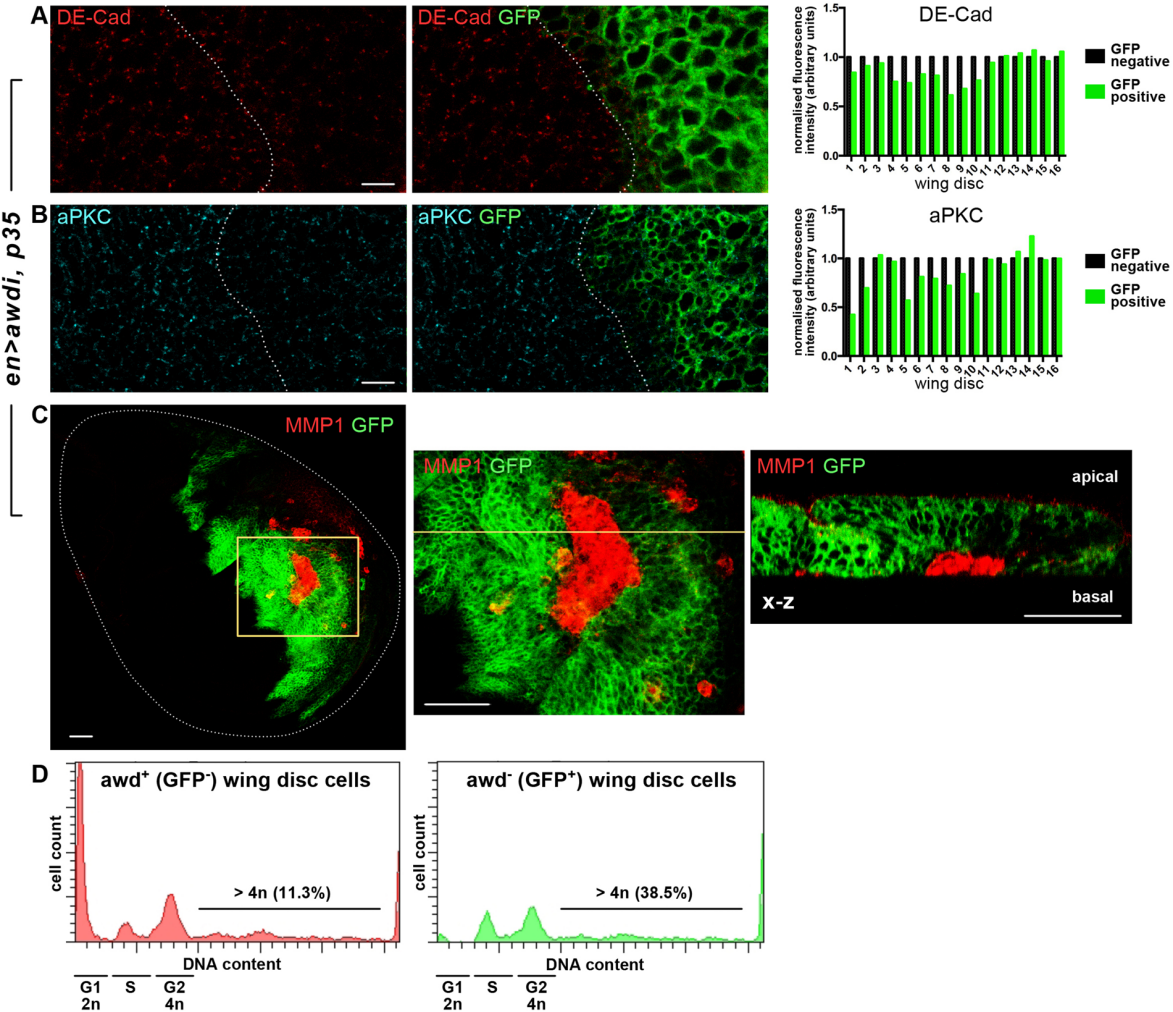
*awd* knockdown leads to CIN. (**A**–**C**) Confocal images of wing discs from *en* > *awdi, p35* third instar larvae. Down-regulation of DE-Cad (**A**) and aPKC stainings (**B**) is detected in posterior compartment cells. To eliminate variability of image intensity among the different wing disc analysed, the mean fluorescence intensity value of GFP positive cells has been normalised to that in GFP negative cells. The histograms show the normalised fluorescence intensity for DE-Cad (**A**) and aPKC (**B**). In the same disc domain, the MMP1 staining (**C**) is greatly up-regulated and the cross section (x-z), along the position indicated by the yellow line, shows that these cells are delaminating. (**D**) Fluorescence associated cell sorter (FACS) of control (GFP^−^) and *awd* depleted cells (GFP^+^) from *en* > *awdi*, *p35* wing discs. The dotted line outlines the anterior/posterior boundary (**A**,**B**). Scale bars: 5 μm in (**A**,**B**), 25 μm in (**C**).

To avoid clearing of *awd* depleted cells, and better characterise the CIN events occurring in those cells, we have co-expressed *awdi* and *p35* under control of *en* driver. Block of apoptotic machinery through expression of the effector caspase inhibitor p35^23^ leads in fact to persistence of CIN cells in tissues. These cells show altered plasma membrane polarity, defects in adherens junctions that connect cells and separate apical and basolateral cellular domains, and delaminate from the epithelium^8^. Then, we have analysed the effects of *awdi* and *p35* co-expression on polarity of wing disc cells (hereafter referred as *en* > *awdi, p35*). We have focused on two proteins, the DE-cadherin (DE-Cad) component of adherens junctions and the atypical protein kinase C (aPKC) component of a sub-apical protein complex^24^. Wing discs were dissected from *en* > *awdi, p35* larvae and in 10 out of 16 discs analysed we detected a visible reduction of DE-Cad (Fig. 3A) and aPKC levels (Fig. 3B). The differences in mean fluorescence intensity between GFP negative and GFP positive cells in each of the 16 wing discs have been analysed using a paired *t*-test. Applying this test the reduction of De-Cad and aPKC stainings in GFP positive cells is statically significant (**p = 0.0081, for DE-Cad and *p = 0.0158, for aPKC) (Supplementary Data S1). Furthermore, *en* > *awdi*, *p35* wing discs show strong up-regulation of MMP1 expression (Fig. 3C, n = 20). High levels of MMP1 lead to basement membrane degradation and cellular invasiveness in normal and tumoural tissues^19,25^. Interestingly, scanning across the vertical axis (x-z section in Fig. 3C) the posterior region of *en* > *awdi*, *p35* wing discs shows that cells with high MMP1 level localise on the basal side of the epithelium. Thus, persistence of *awd* depleted cells in wing disc leads to CIN biological responses including delamination from the epithelium.

CIN consists of gain or loss of whole chromosomes or chromosomal regions leading to cellular aneuploidy. Thus, we have analysed the DNA content profile of cells from the posterior compartment of *en* > *awdi, p35* wing discs using as control cells from the anterior compartment (Fig. 3D). Up to 38.5% of *awdi* cells has DNA content higher than 4n while only 11.3% control cells has a similar DNA content. Since it has been shown that wing disc cells expressing p35 have a percentage of cells with DNA content similar to wild type cells^8^, these results clearly show that *awdi* cells are aneuploid.

## Discussion

**Figure 4:**
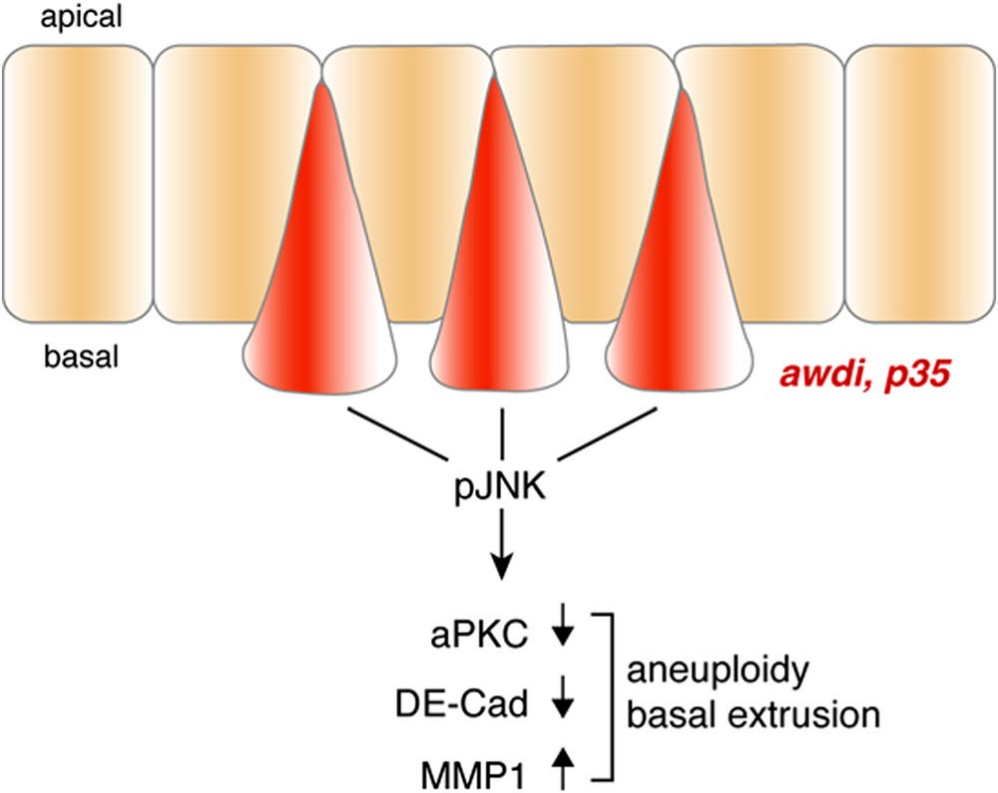
Schematic illustration depicting the consequences of CIN-induced aneuploidy in wing disc cells depleted of *awd* and unable to undergo apoptosis.

We report here, for the first time, a novel role for the Awd endocytic mediator in maintenance of genomic stability in larval disc epithelium. Our results show that depletion of Awd triggers JNK-mediated cell death of wing disc cells and that blocking the cell death machinery results in aneuploidy and cell delamination without overt hyperproliferative effect (Fig. 4). Overgrowth of wing disc hosting aneuploid cells is due to activation of the JNK pathway that promotes expression of Wingless (Wg) upon block of apoptotic cell death. Wg is a mitogenic molecule required in the imaginal discs for growth and patterning^26^ and its expression in the aneuploid, delaminating CIN cells triggers growth of neighbouring non-delaminating cells^8^. However, *awd* ^*J2A4*^ mutant wing disc cells do not express Wg as consequence of faulty Notch signalling^13^ therefore, they cannot promote hyperplasia of the surrounding tissue. Furthermore, lack of hyperproliferation is also observed when aneuploid condition arises from impaired activity of genes controlling karyokinesis. The *diaphanous* gene (*dia*) codes for an actin-regulatory molecule which is required during acto-myosin driven contraction of metaphase furrows^27^. Simultaneous depletion of *dia* gene expression and block of apoptosis do not lead to hyperplastic growth probably due to defective karyokinesis^8^. Intriguingly, Awd is a microtubule-associated nucleoside diphosphate kinase that converts GDP to GTP and the analysis of *awd* mutant larval brain showed mitotic defects correlated with defective microtubule polymerisation^28^. This raises the possibility that the Awd kinase function plays a role in GTP supply to protein such as Orbit which are required for stabilisation of spindle microtubules^29^.

Two lines of evidence further support the hypothesis that Awd could be involved in karyokinesis. The first comes from studies showing that endosome trafficking and transport to the intercellular bridges of dividing cells plays a critical role during abscission, the last step of karyokinesis^30^. In addition, remodelling of plasma membrane that underlies nuclear divisions in syncytial embryo and cellularisation also requires endocytosis^31,32^. Embryo cellularisation requires the dynamin encoded by the *shibire* (*shi*) locus and Rab5 GTPase function since loss of function of either genes arrests ingression of metaphase furrows^33^. Awd functionally interacts with *shi* locus^12^ and it is also required for Rab5 function in early endosomes^13,14^. Thus, a possible role for Awd in cytokinesis should be considered.

The second line of evidence comes from studies on *NME1*, the human homolog of *awd* gene. This metastasis suppressor gene^34^ shares about 78% of aminoacid identity with the *awd* gene^28^. Down-regulation of *NME1* gene expression in diploid cells results in cytokinesis failure and leads to tetraploidy^16^. Our *in vivo* results show that Awd plays a role in maintenance of genomic stability confirming the high degree of conservation between NME1 and Awd proteins. *Drosophila* studies have already been crucial in identification of NME1 function in epithelial morphogenesis and our present work shows that it can be a useful model to investigate also this function and its impact on tumour development and progression.

## Methods

### Fly strains and husbandry

Stocks were raised on standard cornmeal/yeast/agar medium at 25 °C, and crosses were carried out at the same temperature. We used the following Bloomington stocks: #5072, #5073, #30564, #33712, #5137. The stock *ey-Gal4/TM6B* was a gift from A. Giangrande (USIAS, Universitè de Strasbourg); *awd* ^*J2A4*^, *FRT82B*/TM6B (from T. Hsu, Boston University) and *en-Gal4*, *UAS-GFPmCD8*/CyO from D. Grifoni (University of Bologna). Larvae *en-Gal4, UAS-GFPmCD8/*+; *UAS-awdRNAi* and *en-Gal4/UAS-P35; UAS-awdRNAi* were obtained by crossing the parental strains.

### Immunofluorescence Microscopy

Larval tissues were collected and treated at 110–120 hours after egg deposition. Larvae were dissected in 1xPBS at room temperature and fixed for 20 minutes in 4% formaldehyde and the immunostaining procedure was performed as previously described^35^. After several washes in 1xPBS+0,3% Triton X-100, wing imaginal discs were mounted on microscopy slides with Fluoromount G. Subsequently, samples were analysed with TCS SL Leica confocal system. Digital images were assembled using the Adobe Photoshop software. The following primary antibodies were used: monoclonal mouse anti-ci 1:50 (DSHB) and anti-phosphoJNK 1:400 (Cell Signaling Technology) were detected with Cy3-conjugated goat anti-mouse 1:500 (Jackson); polyclonal rabbit anti-Awd^12^ 1:1000 was detected using Cy3-conjugated goat anti-rabbit 1:1000 (Jackson) or DyLight 647-conjugated goat anti-rabbit 1:500 (Jackson); rabbit anti-MMP1 1:50 (DSHB) was detected using Cy3-conjugated goat anti-rabbit 1:1000 (Jackson); anti-cleaved-Caspase3 1:100 (Cell Signaling Technology) was detected using Cy3-conjugated goat anti-rabbit 1:2000 (Sigma); anti-ξPKC 1:200 (Santa Cruz Biotechnology) was detected with Cy5-conjugated goat anti-rabbit 1:1000 (Jackson). DE-Cad 1:25 (DSHB) was detected using Cy3-conjugated goat anti-rat 1:1000 (Jackson).

### Statistical analyses

Fluorescence stains have been captured on TCS SL Leica confocal microscope. Five stacks *per* wing disc have been acquired with xzy scaling of 630 μm. Fluorescence intensity quantification of projected z-stack images has been restricted to neighbour areas of same size in anterior and posterior compartments. Image J software has been used for measurement of fluorescence intensity. Differences between mean fluorescence intensity have been estimated for statistical significance using a two-tailed distribution paired Student’s *t*-test with Prism software.

### Scanning Electron Microscopy

ey-Gal4/UAS-awdRNAi and ey-FLP; Act-Gal4, UAS-GFP; awd^J2A4^, FRT82B/Tub-Gal80, FRT82B and control (ey-Gal4/UAS-GFP and ey-FLP; Act-Gal4, UAS-GFP; Tub-Gal80, FRT82B) flies were collected and washed several times in water and then dehydrated in 100% ethanol. Flies were then incubated in a solution of ethanol:tetramethylorthosilicate (TMOS) 1:1 for 2 hours and then let dry in TMOS 100% overnight at room temperature in a fume hood. The day after the heads were dissected and carefully mounted on an aluminium stub previously prepared with a double stick carbon tape. Samples were then coated with a gold film before SEM examination. Samples were analysed with SEM JEOL JSM-5400 microscope and images were recorded at accelerating voltage of 15 kV.

### Mounting of adult wings

The left wings from female flies were washed in 1xPBS, dehydrated in ethanol 100% and then dissected and mounted on glasses in lactic acid/ethanol (6:5). Wing images were captured using a Nikon Eclipse 90i microscope.

### Flow cytometry analysis

Approximately 60 L3 wing discs for genotype were dissected in cold 1xPBS, centrifuged (5 minutes 3000rcf at RT) and dissociated into single cells in 0,05% Trypsin-EDTA (1X) (Gibco) for 4 h in RT. After dissociation, samples were incubated overnight at −20 °C in a solution of ethanol:PBS 2.5:1. After several washes in 1xPBS+EDTA, samples were incubated with propidium iodide, PI (15 minutes) and analysed by FACS.

PI fluorescence was determined by flow cytometry using a Fluorescent activated cell sorter BD FACSaria Cell Sorter (BD Bioscences). Excitation of the sample was carried out using a Coherent Sapphire Solid State laser. Excitation with 488 nm allowed the acquisition of forward-scatter (FS), side-scatter (SS), fluorescence from GFP. Doublets were discriminated using an integral/peak dot plot of PI fluorescence. Optical alignment was based on optimized signal from specialised fluorescent 6 μm particle (BD AccuDrop beads). DNA analysis on single fluorescence histograms was done using BD FACSDiVa software (BD Biosciences).

## Electronic supplementary material

Supplementary Data S1

## References

[1] Aguilera A, Garcia-Muse T (2013). Causes of genome instability. Annu Rev Genet.

[2] Holland AJ, Cleveland DW (2009). Boveri revisited: chromosomal instability, aneuploidy and tumorigenesis. Nat Rev Mol Cell Biol.

[3] Andriani GA, Vijg J, Montagna C (2017). Mechanisms and consequences of aneuploidy and chromosome instability in the aging brain. Mech Ageing Dev.

[4] Geigl JB, Obenauf AC, Schwarzbraun T, Speicher MR (2008). Defining ‘chromosomal instability’. Trends Genet.

[5] Thompson SL, Bakhoum SF, Compton DA (2010). Mechanisms of chromosomal instability. Curr Biol.

[6] Lara-Gonzalez P, Westhorpe FG, Taylor SS (2012). The spindle assembly checkpoint. Curr Biol.

[7] Liu D, Shaukat Z, Hussain R, Khan M, Gregory SL (2014). Drosophila as a model for chromosomal instability. AIMS Genetics.

[8] Dekanty A, Barrio L, Muzzopappa M, Auer H, Milan M (2012). Aneuploidy-induced delaminating cells drive tumorigenesis in Drosophila epithelia. Proc Natl Acad Sci USA.

[9] Wong HW, Shaukat Z, Wang J, Saint R, Gregory SL (2014). JNK signaling is needed to tolerate chromosomal instability. Cell Cycle.

[10] Nallamothu G, Dammai V, Hsu T (2009). Developmental function of Nm23/awd: a mediator of endocytosis. Mol Cell Biochem.

[11] Nallamothu G, Woolworth JA, Dammai V, Hsu T (2008). *awd*, the homolog of metastasis suppressor gene *Nm23*, regulates *Drosophila* epithelial cell invasion. Mol Cell Biol.

[12] Dammai V, Adryan B, Lavenburg KR, Hsu T (2003). *Drosophila awd*, the homolog of human *nm23*, regulates FGF receptor levels and functions synergistically with *shi/dynamin* during tracheal development. Genes Dev.

[13] Ignesti M (2014). Notch signaling during development requires the function of awd, the Drosophila homolog of human metastasis suppressor gene Nm23. BMC Biol.

[14] Woolworth JA, Nallamothu G, Hsu T (2009). The *Drosophila* metastasis suppressor gene *Nm23* homolog, *awd*, regulates epithelial integrity during oogenesis. Mol Cell Biol.

[15] Hsu T (2011). NME genes in epithelial morphogenesis. Naunyn Schmiedebergs Arch Pharmacol.

[16] Conery AR, Sever S, Harlow E (2010). Nucleoside diphosphate kinase Nm23-H1 regulates chromosomal stability by activating the GTPase dynamin during cytokinesis. Proc Natl Acad Sci USA.

[17] Milan M, Clemente-Ruiz M, Dekanty A, Muzzopappa M (2014). Aneuploidy and tumorigenesis in Drosophila. Semin Cell Dev Biol.

[18] Eaton S, Kornberg TB (1990). Repression of ci-D in posterior compartments of Drosophila by engrailed. Genes Dev.

[19] Uhlirova M, Bohmann D (2006). JNK- and Fos-regulated Mmp1 expression cooperates with Ras to induce invasive tumors in Drosophila. EMBO J.

[20] Lai EC, Rubin GM (2001). Neuralized is essential for a subset of Notch pathway-dependent cell fate decisions during Drosophila eye development. Proc Natl Acad Sci USA.

[21] del Valle Rodriguez A, Didiano D, Desplan C (2011). Power tools for gene expression and clonal analysis in Drosophila. Nat Methods.

[22] Newsome TP, Asling B, Dickson BJ (2000). Analysis of Drosophila photoreceptor axon guidance in eye-specific mosaics. Development.

[23] Hay BA, Wolff T, Rubin GM (1994). Expression of baculovirus P35 prevents cell death in Drosophila. Development.

[24] Tepass U, Tanentzapf G, Ward R, Fehon R (2001). Epithelial cell polarity and cell junctions in Drosophila. Annu Rev Genet.

[25] Srivastava A, Pastor-Pareja JC, Igaki T, Pagliarini R, Xu T (2007). Basement membrane remodeling is essential for Drosophila disc eversion and tumor invasion. Proc Natl Acad Sci USA.

[26] Giraldez AJ, Cohen SM (2003). Wingless and Notch signaling provide cell survival cues and control cell proliferation during wing development. Development.

[27] Afshar K, Stuart B, Wasserman SA (2000). Functional analysis of the Drosophila diaphanous FH protein in early embryonic development. Development.

[28] Biggs J, Hersperger E, Steeg PS, Liotta LA, Shearn A (1990). A Drosophila gene that is homologous to a mammalian gene associated with tumor metastasis codes for a nucleoside diphosphate kinase. Cell.

[29] Inoue YH (2000). Orbit, a novel microtubule-associated protein essential for mitosis in Drosophila melanogaster. J Cell Biol.

[30] D’Avino PP, Capalbo L (2016). Regulation of midbody formation and function by mitotic kinases. Semin Cell Dev Biol.

[31] Rikhy R, Mavrakis M, Lippincott-Schwartz J (2015). Dynamin regulates metaphase furrow formation and plasma membrane compartmentalization in the syncytial Drosophila embryo. Biology Open.

[32] Sokac, A. M. & Wieschaus, E. Local Actin-Dependent Endocytosis Is Zygotically Controlled to Initiate Drosophila Cellularization. *Developmental Cell* **14**, 775–786, 10.1016/j.devcel.2008.02.014. 10.1016/j.devcel.2008.02.014 PMC2517610 18477459

[33] Pelissier, A., Chauvin, J.-P. & Lecuit, T. Trafficking through Rab11 Endosomes Is Required for Cellularization during Drosophila Embryogenesis. *Current Biology* **13**, 1848–1857, 10.1016/j.cub.2003.10.023. 10.1016/j.cub.2003.10.023 14588240

[34] Steeg PS (1988). Evidence for a novel gene associated with low tumor metastatic potential. J Natl Cancer Inst.

[35] Romani P (2016). Dynamin controls extracellular level of Awd/Nme1 metastasis suppressor protein. Naunyn Schmiedebergs Arch Pharmacol.

